# ^1^H-NMR metabolite profiles of different strains of *Plasmodium falciparum*

**DOI:** 10.1042/BSR20140134

**Published:** 2014-11-21

**Authors:** Rongwei Teng, Adele M. Lehane, Markus Winterberg, Sarah H. Shafik, Robert L. Summers, Rowena E. Martin, Donelly A. van Schalkwyk, Pauline R. Junankar, Kiaran Kirk

**Affiliations:** *Research School of Biology, The Australian National University, Canberra, ACT 2601, Australia

**Keywords:** ^1^H-NMR, chloroquine resistance, HPLC, malaria, metabolomics, PfCRT, CQ, chloroquine, CQR, CQ-resistant, CQS, CQ-sensitive, cRBC, co-cultured red blood cell, DV, digestive vacuole, GABA, 4-aminobutyrate, iRBC, infected red blood cell, PfCRT, *Plasmodium falciparum* chloroquine resistance transporter, RBC, red blood cell, TCA, tricarboxylic acid, TSP, trimethylsilyl-2,2,3,3-tetradeuteropropionic acid, uRBC, uninfected red blood cell

## Abstract

Although efforts to understand the basis for inter-strain phenotypic variation in the most virulent malaria species, *Plasmodium falciparum*, have benefited from advances in genomic technologies, there have to date been few metabolomic studies of this parasite. Using ^1^H-NMR spectroscopy, we have compared the metabolite profiles of red blood cells infected with different *P. falciparum* strains. These included both chloroquine-sensitive and chloroquine-resistant strains, as well as transfectant lines engineered to express different isoforms of the chloroquine-resistance-conferring *pfcrt* (*P. falciparum* chloroquine resistance transporter). Our analyses revealed strain-specific differences in a range of metabolites. There was marked variation in the levels of the membrane precursors choline and phosphocholine, with some strains having >30-fold higher choline levels and >5-fold higher phosphocholine levels than others. Chloroquine-resistant strains showed elevated levels of a number of amino acids relative to chloroquine-sensitive strains, including an approximately 2-fold increase in aspartate levels. The elevation in amino acid levels was attributable to mutations in *pfcrt*. *Pfcrt*-linked differences in amino acid abundance were confirmed using alternate extraction and detection (HPLC) methods. Mutations acquired to withstand chloroquine exposure therefore give rise to significant biochemical alterations in the parasite.

## Abstract 2

The metabolite profiles of red blood cells infected with different malaria parasite strains were compared. Amino acid profiles varied with the chloroquine resistance status of the strain, and this was linked specifically to mutations in the parasite's chloroquine resistance transporter.

## INTRODUCTION

Malaria, an ancient disease caused by protozoan *Plasmodium* parasites, continues to inflict a devastating toll in many parts of the world. *Plasmodium falciparum*, the most virulent of the malaria parasite species infectious to humans, has a demonstrated ability to evolve in response to pressures exerted by drug treatments and host immune responses. Many strains of *P. falciparum* have been isolated from different parts of the world and their phenotypes (e.g., drug susceptibility profiles, growth rates and transmissibility) investigated. Work on understanding the molecular basis for important phenotypic differences is underway and has benefited from advances in genomic, transcriptomic and proteomic technologies [1]. Recent years have seen the first reported ‘metabolomic’ analyses of malaria parasite strains [2–5]. However, to date, no untargeted metabolomic comparisons of genetically divergent strains have been reported.

One *P. falciparum* adaptation that represented a significant setback to malaria control efforts was the acquisition of CQ (chloroquine) resistance. The synthetic drug CQ was cheap, safe and unusual in that it remained effective for decades, despite having been deployed as a monotherapy on a massive scale. However, CQR (CQ-resistant) parasites eventually arose in several locations, and they subsequently disseminated throughout most malaria-endemic areas [6,7], rendering CQ largely ineffective.

CQ resistance is associated with mutations in PfCRT (*P. falciparum* chloroquine resistance transporter) [8], a 424 amino acid integral membrane protein. PfCRT localizes to the membrane of the parasite's internal DV (digestive vacuole) [8,9], an acidic organelle in which haemoglobin endocytosed from the host RBC (red blood cell) is catabolized, releasing peptides and haem. CQ accumulates to high concentrations in this compartment through a combination of weak-base trapping and haem binding [10], and is believed to kill the parasite by inhibiting the process by which toxic haem monomers are converted into inert haemozoin crystals [11]. Expression of wild-type PfCRT and a mutant form of the protein (from the CQR Dd2 strain) in *Xenopus laevis* oocytes revealed that the mutant protein mediates CQ transport, whereas the wild-type protein does not [12]. This is consistent with previous biochemical studies that had provided indirect evidence for mutant PfCRT-mediated CQ efflux from the DV [13–15], and explains (at least in part) the reduced intravacuolar CQ accumulation observed in parasites with mutant PfCRT [10].

Much remains to be learned about what impact PfCRT mutations have on the parasite, beyond enabling it to withstand higher CQ concentrations. It is clear that mutations in PfCRT affect the susceptibility of the parasite to a number of other antimalarial drugs [16] and to a diverse range of other compounds [17], and there is indirect evidence for the transport of a range of drugs and ‘chloroquine resistance reversers’ by mutant forms of the protein [18,19]. Moreover, a number of these drugs and compounds have been shown to interact with mutant PfCRT in the *Xenopus* oocyte system (reviewed in [20]). This suggests that mutations in PfCRT have broadened the substrate specificity of the transporter well beyond that required to accommodate CQ. Furthermore, there are differences in the proteomes [21] and gene expression levels [22] of CQS (CQ-sensitive) and CQR parasites, suggesting that PfCRT mutations may have wide-reaching impacts on parasite biology. Attempts to disrupt the *pfcrt* gene have been unsuccessful [23], consistent with PfCRT performing a function that is essential for parasite survival. It remains unclear what this function is, or what the natural substrate(s) of PfCRT might be.

In this study, we have used a combination of ^1^H-NMR and HPLC analyses to compare metabolite profiles of uRBCs (uninfected RBCs) and RBCs infected with different strains of *P. falciparum*. The analyses included parasites expressing different isoforms of PfCRT, allowing us to gain insight into the biochemical consequences of mutations in this transporter.

## EXPERIMENTAL

### Cells and techniques

Seven strains of *P. falciparum* were examined in this study. 3D7 (isolated in the Netherlands but possibly of African origin [24]) and D10 (from Papua New Guinea) are laboratory-adapted CQS strains, while 7G8 (from Brazil) and K1 (from Thailand) are laboratory-adapted CQR strains. C2^GC03^, C4^Dd2^ and C6^7G8^ (a gift from Professor David Fidock) are transfectant lines generated using GC03 [16], a CQS progeny of the HB3×Dd2 cross [25]. The C4^Dd2^ and C6^7G8^ lines were generated by replacing the wild-type *pfcrt* allele in GC03 with the CQ resistance-conferring mutant alleles of *pfcrt* from the Dd2 strain (of Thai origin) and the 7G8 strain, respectively. C6^7G8^ contains an additional I351 M mutation in PfCRT that does not occur in 7G8 parasites [18]. The C2^GC03^ line is a CQS recombinant control line that expresses the wild-type *pfcrt* coding sequence.

The use of human blood in this study was approved by the Australian National University's Human Research Ethics Committee.

Parasites were cultured as described previously [26], and synchronized by sorbitol treatment [27]. The ‘parasite culture medium’ consisted of RPMI supplemented with HEPES (25 mM), additional glucose (10 mM, giving a final concentration of 20 mM), gentamicin sulfate (24 mg l^−1^), hypoxanthine (0.2 mM) and Albumax II (0.6% w/v). The *pfcrt* transfectant lines were maintained in the presence of the selection agents blasticidin (5 μM; Life Technologies) and WR99210 (5 nM; Jacobus Pharmaceuticals). Experiments were conducted on mature intact trophozoite-iRBCs (infected RBCs).

iRBCs were separated from the uninfected cells present in the cultures [henceforth referred to as cRBCs (‘co-cultured’ RBCs)] using a magnet (either a VarioMACS Separator with a CS column or a SuperMACS II Separator with a D column; Miltenyi Biotec), essentially as described previously [5]. Briefly, the cells were centrifuged and resuspended in PBS (pH 7.4) supplemented with 0.5% (w/v) Albumax II and 20 mM glucose immediately before being passed through a column placed within the separation unit's magnetic field. The iRBCs were retained on the column whereas the cRBCs flowed through. After the cRBCs were eluted (with the solution described above), the iRBCs (>95% parasitaemia for NMR analysis; ≥92% for HPLC analysis) were recovered from the column. For experiments on cRBCs, ~3 ml of eluate was collected from the column, while it was in the magnetic field. There was <5% contamination of this predominantly cRBC suspension with iRBCs. For experiments on uRBCs that had been cultured (for ~48 h at 37°C in parasite culture medium) in separate flasks not containing iRBCs (henceforth referred to as uRBCs), the cells were also passed through the column placed within the magnetic field and the eluate collected.

After passing through the column the cells (iRBCs, cRBCs or uRBCs) were, in all cases, resuspended (typically at a density of 1–4×10^8^ cells/50 ml) in parasite culture medium, and were allowed to recover at 37°C, typically for 1 h, but in some cases for up to 4 h. HPLC experiments using iRBCs sampled 1, 2, 3 and 5.5 h post-magnet-enrichment revealed that amino acid levels remained approximately constant throughout this time period (results not shown). This suggests that a 1 h recovery time was adequate and, furthermore, that minor differences in the average age of the trophozoite–iRBC population between experiments would not give rise to significant variation in the metabolite levels.

At the end of the recovery period, the cell suspensions (in 50 ml tubes) were cooled to 4°C within 80 s in a dry-ice/ethanol bath, and then centrifuged (700×***g***, 3 min) at 4°C to sediment the cells. The supernatant solutions were aspirated, and the pellets were resuspended in ice-cold PBS (not supplemented with glucose or Albumax II; ~50 ml) before being centrifuged again as above. The washed cells were then resuspended in a known volume (1–2 ml) of ice-cold PBS and a sample (10 μl) was taken for cell counting (using an improved Neubauer counting chamber) before pelleting the cells by centrifugation (5000×***g***, 1 min) at 4°C. The supernatant solutions were removed, and the pellets frozen immediately by transferring to liquid nitrogen. The frozen pellets were stored at −80°C prior to extraction.

### Extraction of metabolites

For the ^1^H-NMR studies, the metabolites were extracted using HClO_4_, which has previously been shown to have advantages over other extraction media [5]. Ice-cold HClO_4_ (5% w/v; 800 μl) was added to each of the frozen pellets and the samples were transferred to an ultrasonic bath for 20 min, with mixing (30 s using a vortex) at 5 and 15 min. At the end of the 20 min the samples were centrifuged (20800×***g***, 10 min) and the supernatant solutions were collected and approximately neutralized by the addition of 10 M KOH. The samples were then centrifuged at 20800×***g*** for 10 min to remove the precipitated KClO_4_. The supernatant solutions were adjusted to approximately pH 7.0 by addition of 1 M HCl or KOH, centrifuged again to remove the precipitate (when necessary) then freeze dried and stored at −20°C until analysis.

For HPLC (performed primarily to analyse amino acid levels in iRBCs), methanol was used for extraction. It has been shown previously that methanol and HClO_4_ extract most amino acids with comparable efficiency [5]. First, the non-naturally occurring amino acid benzylserine (dissolved in methanol) was added to the pellets as a reference compound, in an amount proportional to the number of iRBCs in the sample (3.26 mmol per 10^7^ iRBCs). HPLC-grade methanol (300 μl) was then added. The samples were mixed by vortexing, and then incubated at 70°C for 20–25 min, with vortexing every ~5 min. They were then centrifuged (13300×***g***, 10 min) and the supernatant solutions were filtered into HPLC vials. The methanol was evaporated using a Genevac miVac Duo concentrator (~100 min) and the samples stored at −20°C prior to derivatization.

### NMR analysis

The freeze-dried extracts were dissolved in a known volume of D_2_O (500–600 μl) buffered at pH 7.0 with 100 mM phosphate, with TSP (trimethylsilyl-2,2,3,3-tetradeuteropropionic acid) added at a concentration of 0.145 mM. TSP was used both as an internal chemical shift reference (0 ppm) and as an intensity reference. The pH of the dissolved NMR samples was further adjusted to neutral when necessary, using small volumes of DCl or NaOD (<5 μl). The samples were centrifuged for 5 min at 16100×***g*** to remove any insoluble material, and 490–550 μl of the supernatant solutions were transferred to Wilmad NMR tubes (5 mm OD) for ^1^H-NMR analysis.

The ^1^H-NMR spectra were acquired and processed essentially as described previously [5], with 16–128 free induction decays collected over a spectral width of 12 ppm and an acquisition time of 4 s, with a 1 s relaxation delay. Concentrations of metabolites in the NMR samples were estimated by importing the spectra into the Chenomx NMR suite (4.6 or 5.0) [28]. This software uses the known concentration of a reference compound (TSP in this study) to determine the concentrations of other compounds. The Chenomx NMR program, to which a number of compounds had been added previously [5] and to which 2,3-bisphosphoglycerate (5 mM with TSP at 1 mM) was added for the purpose of this study, was able to fit almost all of the peaks in the spectra. The identity of sorbitol was confirmed by addition of an authenticated standard. The peak assignments for myoinositol and acetate were confirmed in 2D NMR [HSQC (heteronuclear single-quantum coherence)] analyses of parasite extracts, as described in our previous study [5]. The peak assignments for sarcosine and formate were reliant on a limited number of spectral signals and should be regarded as tentative. The signal-to-noise ratios of the lowest intensity signals from which metabolite concentrations were estimated were typically>6 (Supplementary Figure S1).

### HPLC analysis

The freeze-dried extracts were dissolved in 95 μl of 100 mM sodium bicarbonate (in water, pH 9) and sonicated for 30 s to ensure complete solubilization of the pellets. A 5-μl aliquot of 10 mM TNBS (2,4,6-trinitrobenzenesulfonic acid, used to derivatize, and thus allow HPLC-detection of compounds containing amino groups [29]) in methanol was added and the samples were then incubated for 2 h at 37°C.

The derivatized samples were analysed using a Dionex Ultimate 3000 RSLC system. The analytes were separated on a Grace Vision HT C18 HL column (1.5 μm, 2.0×100 mm) at 35°C, using a flow rate of 0.5 ml/min with 100 mM ammonium acetate in water, pH 7 (A), and acetonitrile (B) as the mobile phase. The mobile phase gradient was set up as follows: 0 min: 90% A and 10% B, 10 min: 50% A and 50% B, 12–14 min: 100% B, 15–20 min: 90% A and 10% B. The derivatized compounds were detected at 335 nm using a Dionex Diode Array Detector.

### Measurements of radiolabelled aspartate and CQ uptake in *X. laevis* oocytes expressing PfCRT or rat GLAST (glutamate–aspartate transporter)

*In vitro* transcription, oocyte preparation and microinjection of PfCRT cRNA (20 ng per oocyte) and GLAST cRNA (5 ng per oocyte) were performed as described elsewhere [12,30]. GLAST cRNA was a gift from Professor Stefan Bröer [31]. [^3^H]CQ (0.25 μM; 20 Ci/mmol) and [^14^C]L-aspartate (6.4 μM; 217 mCi/mmol) uptake by oocytes was measured (3–5 days post-injection) as described previously [30] over 2 h at 27.5°C, and in a medium that, unless specified otherwise, contained 96 mM NaCl, 2 mM KCl, 1 mM MgCl_2_, 1.8 mM CaCl_2_, 10 mM MES and 10 mM Tris-base (pH 5.5). [^3^H]CQ uptake was measured in the presence of 15 μM unlabelled CQ.

## RESULTS

### ^1^H-NMR estimates of metabolite amounts in uRBCs and *P. falciparum*-iRBCs

In initial experiments, the metabolite profile of iRBCs (infected with 3D7 parasites) was compared with that of cRBCs cultured in the same flask, as well as with that of uRBCs (from the same batch) cultured concurrently in a separate flask. Six independent experiments were performed with O+ blood from four different donors, with the blood having been stored at 4°C for 6–23 days prior to use.

**Figure 1:**
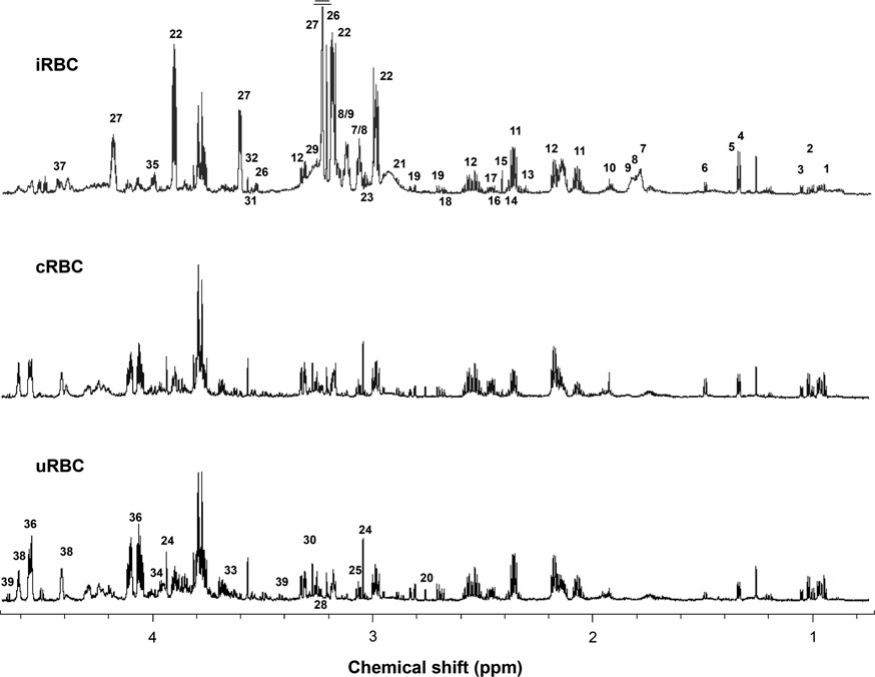
Representative ^1^H-NMR spectra of HClO_4_ extracts of iRBCs (top), cRBCs (middle) and uRBCs (bottom) 1. Leucine, 2. Isoleucine, 3. Valine, 4. Lactate, 5. Threonine, 6. Alanine, 7. Putrescine, 8. Spermidine, 9. Spermine, 10. Acetate, 11. Glutamate, 12. Oxidized glutathione, 13. 4-Aminobutyrate, 14. Pyruvate, 15. Succinate, 16. α-Ketoglutarate, 17. Glutamine, 18. Malate, 19. Aspartate, 20. Sarcosine, 21. Asparagine, 22. HEPES, 23. Lysine, 24. Creatine, 25. Ornithine, 26. Choline, 27. Phosphocholine, 28. Carnitine, 29. Arginine, 30. Betaine, 31. *myo*-Inositol, 32. Glycine, 33. Sorbitol, 34. Serine, 35. Phosphoethanolamine, 36. 2,3-Bisphosphoglycerate, 37. NAD^+^, 38. ATP, 39. Glucose. Other metabolites listed in Table 1 are not in the region shown. The horizontal bar in the top panel signifies that the peak was truncated.

Representative ^1^H-NMR spectra of HClO_4_ extracts derived from iRBCs, cRBCs and uRBCs are shown in Figure 1. Overall, more than 40 metabolites were identified in the ^1^H-NMR analysis. These included amino acids, GSSG, nucleotides, soluble phospholipid precursors, carboxylates, polyamines and HEPES (Table 1). GSH was not detected, consistent with it having been oxidized to GSSG during the preparation of samples for NMR analysis, as noted previously [5]. Glucose was not detected in the 3D7 iRBCs and the amount detected in uRBCs and cRBCs was highly variable. Low levels of glucose were detected in some, but not all, of the extracts obtained from RBCs infected with other parasite strains (see Table 3); again, the amounts detected were highly variable. The absence of glucose from most, although not all, samples, and the high degree of variability seen, is most likely due to the rapid loss of glucose during sample preparation. Glucose is unusually susceptible to loss during sample preparation because: (i) it traverses the parasite and erythrocyte plasma membranes extremely rapidly (on a timescale of a few seconds under physiological conditions [5,32]); and (ii) in the case of iRBCs, it is consumed by the parasite's primary means of energy generation, glycolysis, at a very high rate. Although the cells were cooled to 4°C before processing, and this will have slowed both transport and metabolism, there will have been residual levels of both activities operating throughout the sample preparation procedure, leading to the loss of glucose.

**Table 1:** Metabolite amounts in uRBCs, cRBCs and RBCs infected with 3D7 trophozoites (iRBCs) The metabolite amounts (μmol per 10^12^ cells; obtained by ^1^H-NMR) are the means (shown ±S.D.) from 6 independent cell preparations (obtained on different days). The cRBC preparations contained up to 5% contamination with iRBCs and the iRBC preparations had up to 5% contamination with cRBCs. Depending on the metabolite, the amounts presented here are all within 95%-109% of the values that would have been obtained had the cell preparations been 100% pure. nd, not detected in some or all replicates. a*P*<0.001, ^b^*P*<0.01 and ^c^*P*<0.05, compared to uRBC, from one-way ANOVA tests using the natural logarithm transformed data (nd data excluded). The *P* values were corrected for multiple hypothesis testing using the False Discovery Rate (FDR) method of Benjamini and Hochberg [51]. Red shading: more abundant in iRBCs than in uRBCs; green shading: less abundant in iRBCs than in uRBCs.

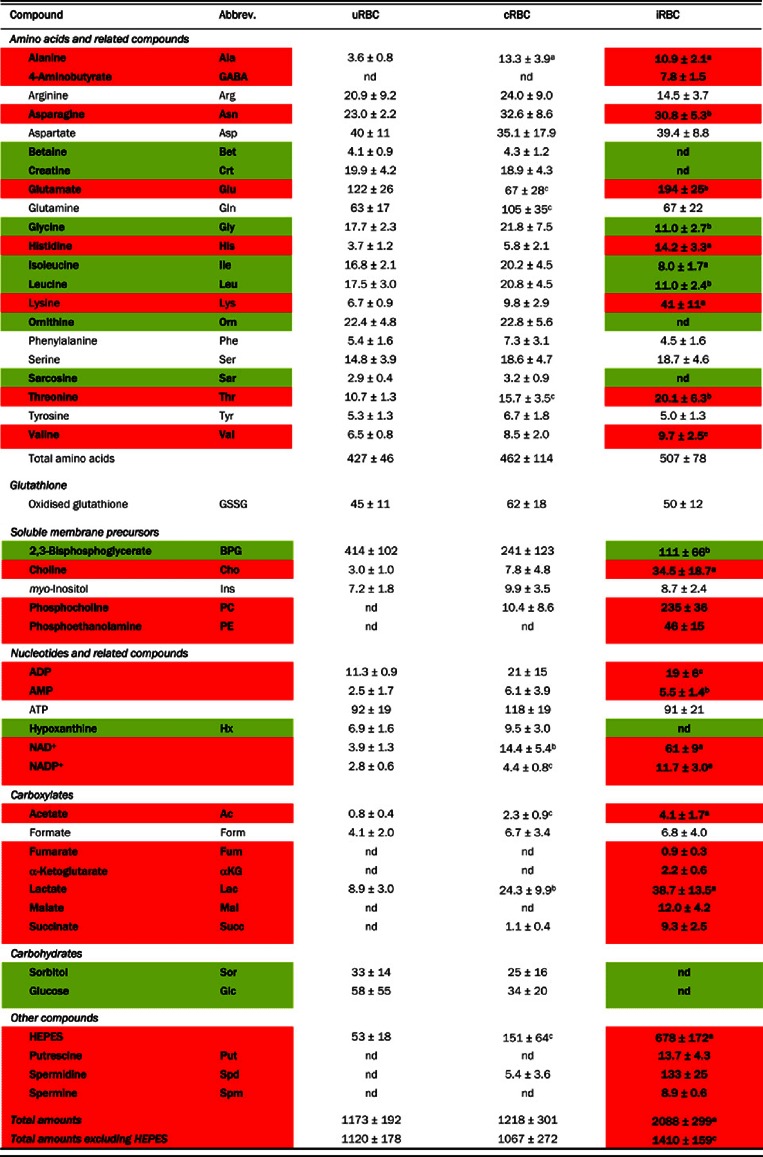

Calculation of the ‘energy charge ratio’ [(ATP+0.5 ADP)/(ATP+ADP+AMP)] using the averaged data from Table 1 yielded values of 0.92, 0.89 and 0.87 for uRBCs, cRBCs and iRBCs, respectively. These all fall within the normal range (0.85–0.95) for cells dependent on glycolysis [33], consistent with the cells having been in good condition prior to extraction.

The metabolite profiles of uRBCs and cRBCs were similar to one another, but quite different from the metabolite profile of iRBCs (Figure 1 and Table 1). In particular, the γ-amino acid GABA (4-aminobutyrate), the phospholipid precursors phosphocholine and phosphoethanolamine, the TCA (tricarboxylic acid) cycle intermediates fumarate, α-ketoglutarate, malate and succinate, and the polyamines putrescine, spermidine and spermine, were all undetectable by ^1^H-NMR in uRBCs but present in iRBCs. Conversely, betaine, creatine, hypoxanthine, ornithine, sarcosine and sorbitol were detected by ^1^H-NMR in uRBCs and cRBCs but not in iRBCs (note however that ornithine was detectable in iRBCs by HPLC; below).

Some compounds were present in all three cell types, but were significantly more abundant in iRBCs than in either of the uRBC populations (Table 1). HEPES (the most abundant solute in iRBCs) was one such compound, consistent with this exogenous buffering agent (used widely for the *in vitro* culture of malaria parasites) being taken up by the parasite, as shown previously in a study of D10 parasites isolated from their host RBCs [5]. Other compounds significantly more abundant in iRBCs than in uRBCs or cRBCs included the amino acids glutamate, histidine, lysine and threonine, as well as choline, NAD^+^ and NADP^+^ and acetate. Alanine and lactate were elevated in both iRBCs and cRBCs relative to uRBCs. Conversely, isoleucine and leucine were less abundant in iRBCs than in uRBCs and cRBCs. 2,3-Bisphosphoglycerate was significantly less abundant in cRBCs than in uRBCs, and was less abundant still in iRBCs, consistent with a previous study [34].

### ^1^H-NMR estimates of metabolite amounts in RBCs infected with different *P. falciparum* strains

Using ^1^H-NMR the intracellular amounts of 39 metabolites were estimated for a number of different CQS and CQR strains derived originally from field isolates (the CQS strains 3D7 and D10 and the CQR strains 7G8 and K1). The results are presented in Table 2. The ANOVA test identified a range of metabolites for which levels showed significant differences among these genetically divergent strains (Table 2). Choline and phosphocholine in particular showed marked variation between strains, being >30-fold and >5-fold more abundant, respectively, in 3D7 (CQS) and 7G8 (CQR) iRBCs than in D10 (CQS) and K1 (CQR) iRBCs (Table 2). These differences, as well as many others, did not track with the CQ resistance status of the strain, and presumably result from genetic differences other than those in *pfcrt*. We did however observe that a number of different amino acids, as well as the total pool of amino acids, were elevated in both CQR strains compared with both CQS strains.

**Table 2:** Metabolite amounts in RBCs infected with different (non-transfectant) *P. falciparum* strains The amounts (μmol per 10^12^ cells; obtained by ^1^H-NMR) are the means±S.D. from the indicated number of independent cell preparations (obtained on different days). In all cases, the cells were mature trophozoite-infected RBCs. The amounts shown are derived from cell preparations with up to 5% contamination with cRBCs. nd, not detected in some or all replicates. a: *P*<0.001; b: *P*<0.01 and c: *P*<0.05 from FDR-adjusted one-way ANOVA tests using the natural logarithm transformed data (see Table 1; nd data excluded). Metabolites for which there were significant differences in abundance among strains are shown in bold.

	ANOVA	3D7	D10	7G8	K1
No. of independent cultures		10	9	10	8
CQ resistance status		CQS	CQS	CQR	CQR
***Amino acids***					
**Alanine**	**a**	**7.8±1.7**	**11.5±1.8**	**20.4±4.7**	**13.9±3.2**
**4-Aminobutyrate**	**a**	**4.3±2.5**	**34.9±8.9**	**23.2±9.3**	**26.0±7.2**
**Arginine**	**a**	**33.2±16.1**	**69±21**	**50±9**	**76±18**
**Asparagine**	**a**	**29.0±7.7**	**30.0±4.7**	**42±8**	**35.9±6.1**
**Aspartate**	**a**	**54±12**	**50±10**	**117±21**	**185±54**
**Glutamate**	**a**	**166±18**	**237±26**	**245±37**	**238±46**
**Glutamine**	**c**	**58±9**	**54±11**	**50±9**	**44±8**
**Glycine**	**a**	**8.8±3.2**	**11.3±1.7**	**21.7±5.1**	**12.9±2.2**
**Histidine**	**a**	**8.9±3.3**	**9.6±3.3**	**20.3±5.1**	**13.7±2.8**
**Isoleucine**	**c**	**5.9±1.4**	**5.6±1.6**	**8.4±1.6**	**6.3±1.9**
**Leucine**	**a**	**9.7±2.9**	**11.1±2.4**	**17.8±3.6**	**11.4±3.2**
**Lysine**	**a**	**48±22**	**96±20**	**77±13**	**239±64**
**Phenylalanine**	**c**	**2.3±3.2**	**4.4±4.3**	**3.7±3.4**	**9.0±14.9**
**Serine**	**a**	**15.3±4.0**	**12.6±2.5**	**27.3±7.6**	**18.2±3.7**
**Threonine**	**b**	**15.7±3.6**	**18.3±2.4**	**21.8±3.9**	**16.8±3.0**
**Tyrosine**	**a**	**4.4±2.0**	**4.4±1.5**	**6.8±1.3**	**4.1±0.8**
**Valine**	**a**	**7.9±1.7**	**9.3±1.0**	**11.8±2.2**	**8.1±1.0**
**Total amino acids**	**a**	**479±92**	**669±63**	**763±99**	**960±126**
***Glutathione***					
**GSSG**	**c**	**43±10**	**54±10**	**48±7**	**54±9**
***Soluble membrane precursors***					
**2,3-Bisphosphoglycerate**	**b**	**114±29**	**81±25**	**110±21**	**132±18**
**Choline**	**a**	**33.2±11.5**	nd	**52±9**	**1.2±0.8**
***myo*-Inositol**	**b**	**5.4±1.5**	**7.8±1.4**	**7.2±1.6**	**8.1±1.8**
**Phosphocholine**	**a**	**220±51**	**36.7±15.5**	**223±41**	**36.2±14.5**
**Phosphoethanolamine**	**a**	**38.7±11.3**	**89±29**	**82±21**	**60±20**
***Nucleotides and related compounds***					
ADP		14.3±3.1	28.3±12.7	17.9±5.7	19.2±7.7
AMP		nd	4.2±3.5	nd	nd
**ATP**	**b**	**88±16**	**124±46**	**121±20**	**137±17**
NAD^+^		56±9	64±9	60±10	54±5
**NADP^+^**	**c**	**9.9±1.8**	**8.7±1.6**	**7.8±1.2**	**8.5±1.0**
***Carboxylates***					
Acetate		10±6	15±19	10.5±4.3	15±12
Formate		9.9±5.8	14±24	11.5±5.4	15.4±8.4
**Fumarate**	**a**	**0.8±0.6**	**2.6±0.3**	**1.5±0.4**	**1.0±0.6**
α-Ketoglutarate		2.5±1.8	2.6±1.3	3.4±0.6	2.8±1.3
Lactate		31.0±7.6	30±14	40±11	50±34
**Malate**	**a**	**13.8±6.5**	**33.6±7.4**	**20.4±3.4**	**15.4±7.1**
**Succinate**	**a**	**8.5±1.5**	**14.6±2.4**	**9.1±2.6**	**4.9±2.7**
***Other compounds***					
HEPES		540±88	590±80	632±92	544±85
**Putrescine**	**a**	**26.1±8.6**	**51±15**	**30.9±6.2**	**31.2±9.9**
Spermidine		164±22	180±26	165±22	167±19
**Spermine**	**a**	**8.4±2.2**	**12.5±2.0**	**16.1±2.6**	**16.6±2.4**
***Total amounts***	**a**	**1991±170**	**2210±234**	**2532±242**	**2425±194**
***Total amounts excluding HEPES***	**a**	**1450±150**	**1620±172**	**1900±190**	**1881±190**

We also estimated metabolite amounts for three transfectant lines that differ solely with respect to the isoform of PfCRT that they express (the CQS line C2^GC03^ and the CQR lines C4^Dd2^ and C6^7G8^; Table 3). The ANOVA test identified a number of metabolites for which levels showed significant differences between lines (Table 3). We again observed that many individual amino acids, as well as the total pool of amino acids, were elevated in both CQR lines relative to the CQS line. Across Tables 2 and 3, RBCs infected with all the four (transfectant and non-transfectant) CQR strains showed higher amounts of aspartate, asparagine, glycine, leucine and serine than those infected with any of the three CQS strains. The amount of the polyamine spermine was also greater for CQR strains than for CQS strains.

**Table 3:** Metabolite amounts in RBCs infected with mature trophozoite-stage *pfcrt* transfectant parasites The amounts (μmol per 10^12^ cells; obtained with ^1^H-NMR) are from the indicated number of independent cell preparations (obtained on different days), and are shown as means±S.D. The amounts shown are derived from cell preparations with up to 5% contamination with cRBCs. a: *P*<0.001; b: *P*<0.01 and c: *P*<0.05 from FDR-adjusted one-way ANOVA tests using the natural logarithm transformed data (see Table 1). Metabolites for which there were significant differences in abundance among lines are shown in bold.

	ANOVA	C2^GC03^	C4^Dd2^	C6^7G8^
No.of independent cultures		9	8	7
CQ resistance status		CQS	CQR	CQR
***Amino acids***				
**Alanine**	**b**	**15.2±4.5**	**20.8±6.6**	**26.7±3.4**
4-Aminobutyrate		4.7±2.7	4.8±1.4	3.6±1.1
**Arginine**	**c**	**24.8±7.5**	**27.9±6.7**	**16.8±6.6**
**Asparagine**	**a**	**22.6±4.5**	**39.0±8.8**	**42±9**
**Aspartate**	**a**	**54±5**	**105±30**	**102±26**
**Glutamate**	**b**	**163±26**	**148±34**	**105±24**
**Glutamine**	**a**	**44±10**	**66±10**	**79±21**
**Glycine**	**a**	**12.8±2.5**	**24.1±5.8**	**27.2±1.5**
**Histidine**	**b**	**14.4±5.9**	**21.2±5.4**	**25.0±5.2**
**Isoleucine**	**a**	**7.4±1.2**	**10.4±1.2**	**12.3±1.9**
**Leucine**	**a**	**10.7±1.9**	**18.5±3.8**	**24.3±13.3**
Lysine		31.8±7.5	29.1±12.1	28.4±6.0
Phenylalanine		9.0±6.3	8.4±3.6	6.5±2.2
**Serine**	**a**	**13.3±3.0**	**22.3±4.0**	**25.3±4.5**
**Threonine**	**b**	**15.3±2.6**	**20.4±4.4**	**22.4±4.5**
**Tyrosine**	**b**	**4.4±0.7**	**6.5±1.5**	**7.0±1.3**
**Valine**	**b**	**6.9±1.0**	**10.2±2.6**	**9.4±1.0**
**Total amino acids**	**b**	**454±52**	**582±89**	**562±62**
***Glutathione***				
GSSG		43±7	41±6	47±6
***Soluble membrane precursors***				
2,3-Bisphosphoglycerate		122±40	146±39	183±61
Choline		69±14	70±15	80±38
***myo*-Inositol**	**c**	**9.4±6.3**	**9.7±1.3**	**15.2±3.4**
Phosphocholine		301±85	226±93	253±117
Phosphoethanolamine		52±15	49±20	55±23
***Nucleotides and related compounds***				
ADP		18.0±4.5	15.9±2.5	19.7±8.6
ATP		114±19	111±22	127±22
NAD^+^		42±7	35.4±12.7	51±15
NADP^+^		4.0±0.7	4.4±1.2	4.5±0.3
***Carboxylates***				
Acetate		10.3±9.0	7.0±3.8	15.6±8.1
Formate		8.6±6.2	9.1±5.5	8.5±4.8
Fumarate		1.0±0.4	0.8±0.5	1.2±0.6
α-Ketoglutarate		2.5±0.7	3.6±1.1	2.6±1.0
Lactate		36.3±14.5	38.5±10.5	43±11
Malate		13.3±8.7	13.7±3.2	10.7±4.5
**Succinate**	**a**	**7.7±1.5**	**4.0±1.7**	**3.7±0.5**
***Other compounds***				
**HEPES**	**c**	**446±39**	**479±92**	**1024±577**
Putrescine		14.3±3.6	10.0±5.8	9.4±6.0
**Spermidine**	**c**	**144±29**	**101±30**	**87±37**
**Spermine**	**a**	**9.9±1.9**	**22.2±11.6**	**27.2±9.1**
Glucose		6.3±8.5	25.6±31.0	38.7±53.9
***Total amounts***		1927±227	2005±309	2669±851
***Total amounts excluding HEPES***		1481±212	1526±255	1646±283

**Figure 2:**
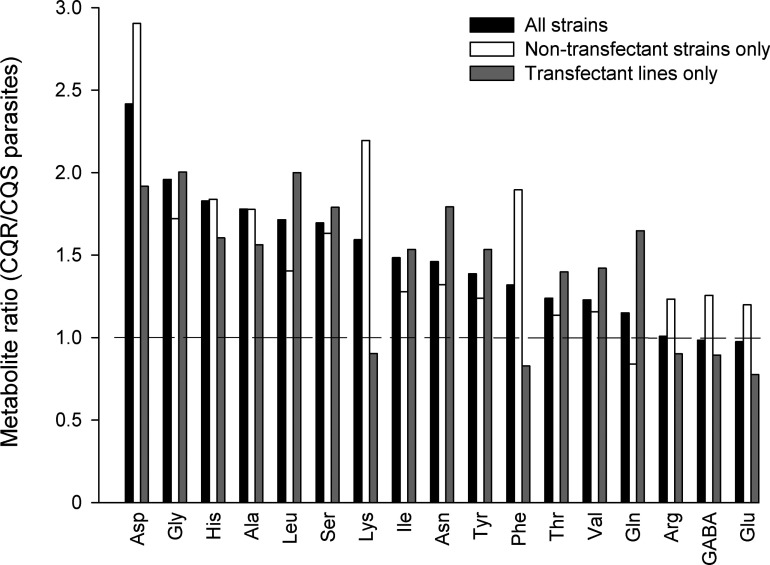
Differences in amino acid amounts in RBCs infected with CQR and CQS parasites Black bars show the mean amino acid amounts for CQR strains (using averaged data for 7G8, K1, C4^Dd2^ and C6^7G8^) divided by those for CQS strains (3D7, D10 and C2^GC03^). The white bars show the mean amino acid amounts for the non-transfectant CQR strains (7G8 and K1) divided by those for the non-transfectant CQS strains (3D7 and D10). The grey bars show the mean amino acid amounts for the transfectant CQR strains (C4^Dd2^ and C6^7G8^) divided by those for the CQS transfectant line C2^GC03^.

Figure 2 shows the ratios of the mean amounts for each (detectable) amino acid in CQR versus CQS strains. In the case of aspartate, the level seen in all CQR strains was >1.8-fold higher than that seen in all CQS strains, with the actual amounts differing by ≥48 μmol per 10^12^ cells in all cases. For the other amino acids that were consistently higher in the CQR strains than the CQS strains the actual amounts were much lower. These differences in amino acid abundance were observed both in genetically disparate CQS (3D7 and D10) and CQR (7G8 and K1) strains derived originally from field isolates (Table 2, Figure 2), and in the transfectant lines that differ solely with respect to the form of *pfcrt* expressed (Table 3 and Figure 2). The results obtained with the transfectant lines allow the differences to be attributed to the differences in *pfcrt*.

### HPLC measurements of metabolites in RBCs infected with different *P. falciparum* strains

**Figure 3:**
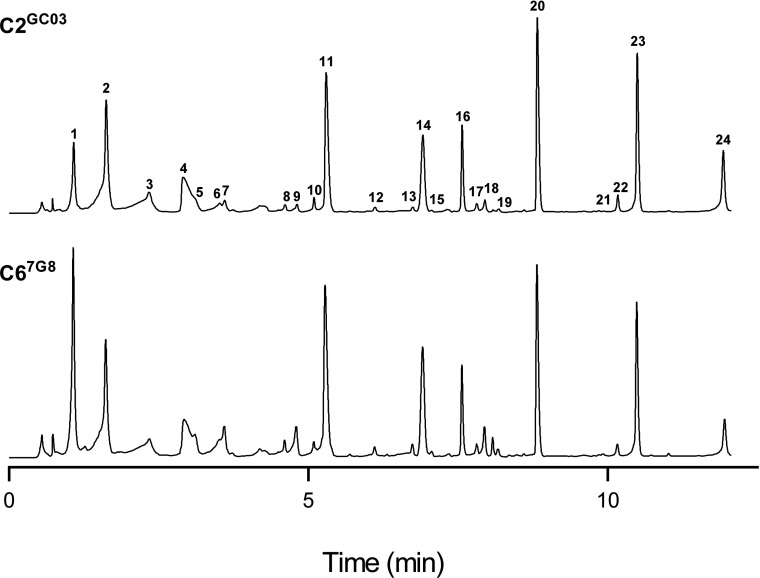
Representative HPLC traces for RBCs infected with C2^GC03^ (top) or C6^7G8^ (bottom) trophozoites 1. Aspartate, 2. Glutamate, 3. Cystine, 4. Background, 5. Asparagine, 6. Glutamine, 7. Serine, 8. Citrulline, 9. Glycine, 10. 4-Aminobutyrate, 11. Alanine, 12. Threonine, 13. Valine, 14. Derivatization artifact, 15. Methionine, 16. Unknown, 17. Isoleucine, 18. Tryptophan, 19. Phenylalanine, 20. Benzylserine (a non-naturally occurring amino acid to which other peaks were normalized), 21. Spermine, 22. Ornithine, 23. Lysine, 24. Spermidine.

The finding that mutations in *pfcrt* give rise to significant differences in amino acid abundance was confirmed using HPLC. The amino acid profiles of the transfectant CQS C2^GC03^ and CQR C6^7G8^ lines were compared using the methanol extraction method, with the extracts subjected to a derivatization procedure to facilitate the detection of compounds containing amino groups. The HPLC-based approach enabled the detection of 18 amino acids and two polyamines. The absolute amounts of these metabolites were not quantified; rather, in each experiment, the metabolite amounts were normalized to that of the non-naturally occurring amino acid benzylserine (added to each sample as a reference compound), allowing the determination of the relative amounts of each amino acid in the two lines. Representative HPLC traces for each line are shown in Figure 3, and the averaged data from three independent cell preparations are presented in Table 4.

**Table 4:** Relative intracellular metabolite amounts obtained by HPLC in RBCs infected with C2^GC03^ or C6^7G8^ trophozoites The data are averaged from three independent cell preparations (obtained on different days) for each line and are shown ±S.E.M. A value >1 indicates that a metabolite is more abundant in C6^7G8^ iRBCs; a value <1 indicates that a metabolite is more abundant in C2^GC03^ iRBCs. nq, not quantified because these compounds were not detected in all experiments (asparagine and spermine were only detected in experiments with C6^7G8^ iRBCs). Red shading: significantly more abundant in C6^7G8^ iRBCs than in C2^GC03^ iRBCs.

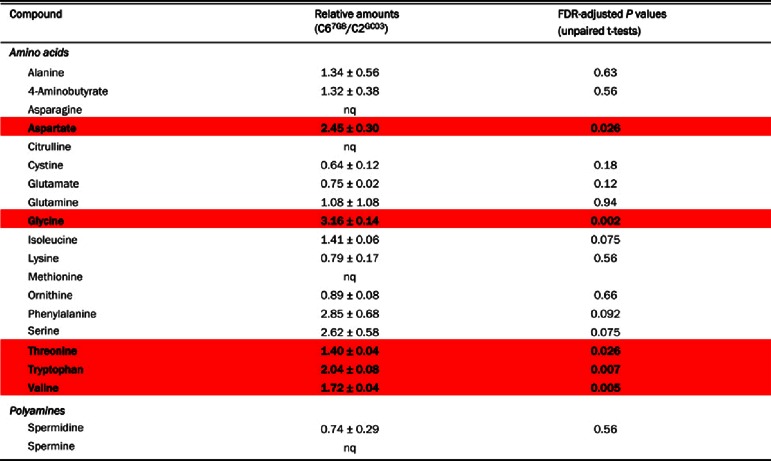

The data obtained with HPLC were, in general, consistent with those obtained with ^1^H-NMR. Of the 20 metabolites detected by HPLC, two (asparagine and spermine) were only detected for C6^7G8^. This is consistent with the ^1^H-NMR data (Table 3), which revealed greater amounts of asparagine and spermine for C6^7G8^ than for C2^GC03^. For each of 13 compounds (12 amino acids+spermidine), the ratio of the amount in the C6^7G8^ iRBCs relative to the amount in C2^GC03^ iRBCs was obtained using both HPLC and ^1^H-NMR. The two methods gave good agreement for the following compounds (although statistical significance was not reached with HPLC in all cases): alanine, aspartate, glutamine, glycine, isoleucine, serine, threonine and valine, all of which were more abundant in C6^7G8^ iRBCs than in C2^GC03^ iRBCs; glutamate and spermidine, both of which were more abundant in C2^GC03^ iRBCs than in C6^7G8^ iRBCs; and GABA and lysine, levels of which did not differ significantly between the two parasite lines. The HPLC measurements indicated a relatively high level of phenylalanine in C6^7G8^ iRBCs compared to C2^GC03^ iRBCs; however, this difference was not statistically significant as there was a high level of inter-experimental variation, most likely as a consequence of the low levels of phenylalanine present in both lines. The ^1^H-NMR measurements also indicated no significant difference between phenylalanine levels in C6^7G8^ and C2^GC03^ iRBCs (Table 3).

HPLC enabled the detection of several metabolites that were not detected by ^1^H-NMR. Citrulline and methionine were not quantified, because they were not detected in all experiments. Tryptophan was present at twice the amount in C6^7G8^ than in C2^GC03^ iRBCs. The amounts of ornithine and the dimeric amino acid cystine did not differ significantly between C6^7G8^ and C2^GC03^ iRBCs.

### Expression of PfCRT in *Xenopus* oocytes

**Figure 4:**
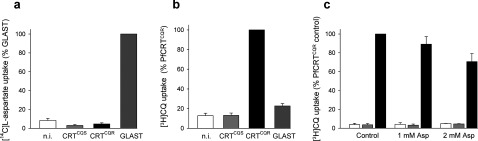
L-Aspartate and CQ uptake by *Xenopus* oocytes expressing PfCRT [^14^C]L-aspartate uptake (**a**) and [^3^H]CQ uptake (**b**) in n.i. (non-injected) oocytes and in oocytes expressing PfCRT^CQS^, PfCRT^CQR^ or rat GLAST. Uptake is shown as the means + S.E.M. from nine separate experiments, within which measurements were made from ten oocytes per treatment. (**c**) Effect of unlabelled L-aspartate (1 and 2 mM) on the uptake of [^3^H]CQ into n.i. oocytes (white bars) and oocytes expressing PfCRT^CQS^ (grey bars) or PfCRT^CQR^ (black bars). Uptake is shown as the means + S.E.M. from three separate experiments, within which measurements were made from ten oocytes per treatment.

One possible explanation for the higher abundance of amino acids in RBCs infected with CQR parasites is that PfCRT normally serves to efflux haemoglobin-derived amino acids from the DV, and that this function is compromised by CQ-resistance-conferring mutations. Using the *Xenopus* oocyte expression system, we tested whether aspartate, the amino acid showing the greatest difference in abundance between CQS and CQR strains, is a substrate of the wild-type (PfCRT^CQS^) or mutant (PfCRT^CQR^) protein. As shown in Figure 4(a), oocytes expressing PfCRT^CQS^ or PfCRT^CQR^ did not accumulate [^14^C]L-aspartate above the levels measured in non-injected oocytes (*P*>0.05; ANOVA with Tukey–Kramer multiple comparisons test), whereas oocytes expressing the rat glutamate/aspartate transporter GLAST showed a marked increase in [^14^C]L-aspartate uptake relative to non-injected oocytes (*P<*0.001). There was no significant difference in [^14^C]L-aspartate uptake between PfCRT^CQS^- and PfCRT^CQR^-expressing oocytes (*P*>0.05). Furthermore, [^14^C]L-aspartate uptake into PfCRT^CQR^ and PfCRT^CQS^-expressing oocytes was not affected by the presence of the PfCRT^CQR^ inhibitors saquinavir (250 μM; [35]), verapamil (250 μM) or CQ (750 μM) [12], or by choline (250 μM), artemisinin (500 μM), or unlabelled L-aspartate (200 μM) (results not shown). In the same experiments, the transport of [^3^H]CQ into oocytes expressing PfCRT^CQR^ was significantly higher than that measured in all other oocyte types (Figure 4b; *P<*0.001).

To investigate further whether there might be an interaction between L-aspartate and PfCRT, we examined whether unlabelled L-aspartate affected the uptake of [^3^H]CQ into *Xenopus* oocytes expressing PfCRT. As shown in Figure 4(c), the addition of 1 mM unlabelled L-aspartate had no significant effect on either the uptake of [^3^H]CQ by oocytes expressing PfCRT^CQR^ or the diffusion of [^3^H]CQ into non-injected oocytes or oocytes expressing PfCRT^CQS^ (*P*>0.05). A modest but significant reduction in [^3^H]CQ uptake was observed in PfCRT^CQR^-expressing oocytes in the presence of 2 mM unlabelled L-aspartate (*P<*0.05), whereas the addition of 2 mM unlabelled L-aspartate had no effect on [^3^H]CQ uptake into non-injected or PfCRT^CQS^-expressing oocytes (*P*>0.05).

## DISCUSSION

Metabolomic comparisons have been applied to great effect in a variety of systems to unravel physiological differences between genders, strains, species and healthy versus diseased tissues. In this study, we have carried out metabolomic comparisons of iRBCs, uRBCs and cRBCs, and of RBCs infected with different strains of *P. falciparum*.

There were significant differences in the abundance of many metabolites between iRBCs and uRBCs, with cRBCs having levels similar to uRBCs for most metabolites (Table 1). As has been noted previously [34], iRBCs have significantly lower levels of 2,3-bisphosphoglycerate than their uninfected counterparts. Glucose was absent from the iRBCs for most of the strains, but present at significant levels in both uRBCs and cRBCs. This difference is likely to be due to intracellular glucose in the iRBCs being consumed by residual glycolysis during the cell isolation procedure; in the uninfected cells glycolysis is two orders of magnitude slower [34] and this will not have been an issue. Sorbitol was also present at significant levels in uRBCs and cRBCs. This is consistent with a previous study in which exposure of uRBCs to elevated extracellular glucose concentrations was found to result in an increased intracellular sorbitol content [36]. The end-product of glycolysis, lactate, was detected in all cell types, but other glycolytic intermediates were below the level of detection. As noted previously, the intraerythrocytic parasite has significant concentrations of TCA cycle intermediates [3,5]. A recent study has provided evidence that a conventional TCA cycle operates at low levels in asexual blood-stage *P. falciparum* parasites [37]. Fumarate and malate were not detected in either uRBCs or cRBCs. The detection of succinate in cRBCs, but not uRBCs may be due to succinate being exported from the parasite and parasitized RBC, to the external medium (from where it may be taken up by cRBCs).

The much higher levels of the membrane precursors choline and phosphocholine in iRBCs than in uRBCs and cRBCs reflects the fact that the parasite, unlike the mature RBC, carries out extensive membrane synthesis [38]. The amounts of choline and phosphocholine differed markedly between iRBCs infected with different strains, being>30-fold and>5-fold more abundant, respectively, in RBCs infected with 3D7 or 7G8 parasites than in those infected with D10 or K1 parasites. The basis for this difference is not known, but may relate to differences in choline uptake between different parasites. There is physiological evidence for a membrane-potential-dependent choline transporter on the parasite plasma membrane [39,40] but its molecular identity (and, therefore, whether there may be polymorphisms in the transporter in some strains) remains to be discovered.

As has been noted previously, there is a significant concentration of the neurotransmitter GABA associated with the parasite [5]. A recent study has reported that TCA cycle intermediates are used to synthesize glutamate, which is then decarboxylated to yield GABA in *P. falciparum* iRBCs [37]. The buffer HEPES, used widely for malaria parasite culture, is also present (and highly abundant) in iRBCs infected with all strains. HEPES was found previously to be an abundant solute in D10 parasites isolated from their host RBCs [5]. The mechanism by which HEPES gains entry into the parasite is not known; given its physicochemical properties, transport proteins are probably involved. The extent to which the uptake of this exogenous agent might affect the physiology of the parasite is yet to be explored. Previous mass spectrometry studies have identified additional metabolites (not detected in this study by ^1^H-NMR) that differ in abundance between uRBCs and *P. falciparum* iRBCs [3,4].

The comparison of the metabolite profiles of RBCs infected with different CQS and CQR parasites, including parasites expressing different *pfcrt* alleles on a single genetic background, uncovered some significant differences. The most striking finding was that the amounts of many individual amino acids, as well as the total amount of amino acids, were elevated in RBCs infected with CQR parasites compared to those infected with CQS parasites. Of particular note was aspartate, which was present in substantially greater amounts for all (non-transfectant and transfectant) CQR strains (102–185 μmol per 10^12^ cells) than for all CQS strains (50–54 μmol per 10^12^ cells). Asparagine, glycine, leucine, serine and the polyamine spermine were also found in greater amounts for all CQR strains than for all CQS strains, though the relative differences were less pronounced. In the *pfcrt* transfectant lines, both the ^1^H-NMR and HPLC analyses indicated higher glycine and serine levels in parasites bearing CQ-resistance-conferring mutations in PfCRT, with the relative differences similar to or greater than that seen for aspartate; however, the amounts involved were substantially lower than was the case for aspartate.

The DV is the site at which the parasite digests the haemoglobin endocytosed from the host RBC. Adult human haemoglobin contains every proteinogenic amino acid except for isoleucine, and haemoglobin digestion is essential for parasite survival [41]. The amino acids produced are used for protein synthesis, and the process might also be important to maintain osmotic balance in the host cell [42] and to ensure that there is sufficient space for parasite growth inside the RBC [43]. The extent to which haemoglobin-derived peptides are broken down within the DV is not yet clear (reviewed in [44]). It is believed that small peptides, including some dipeptides, are generated by proteases located in the DV [45]; however, the localization of exoaminopeptidases that digest small peptides into individual amino acids has been controversial [46–49]. Based on bioinformatic analyses of PfCRT and the requirement that haemoglobin breakdown products be effluxed from the DV, it has been suggested that the natural function of PfCRT might be amino acid and/or peptide transport [50].

One potential explanation for our findings was that (wild-type) PfCRT does indeed transport amino acids out of the DV, and that this function is compromised in part by CQ-resistance-conferring mutations, leading to a build-up of amino acids such as aspartate in this organelle. However, neither PfCRT^CQS^ nor PfCRT^CQR^ mediated aspartate transport when expressed in *Xenopus* oocytes. The finding that 2 mM unlabelled L-aspartate caused a modest decrease in [^3^H]CQ uptake by PfCRT^CQR^-expressing oocytes raises the possibility of an interaction between aspartate and mutant PfCRT. However, any such interaction must be low-affinity and does not result in significant translocation of aspartate.

An alternative possibility is that mutations in PfCRT give rise to the altered amino acid profile observed here via an indirect effect. For example, the build-up of amino acids could result from *pfcrt*-linked changes in DV physiology that affect haemoglobin degradation. Indeed, this was suggested in a recent study in which haemoglobin-derived peptides ranging in size from 2-mers to 32-mers were shown to accumulate in erythrocytes infected with CQR parasites [2]. With the exception of GSSG, peptides fell below the detection limit in our study. Perhaps the small peptides that are proposed to be trapped within the DV of CQR parasites [2] degrade over time to produce amino acids, leading to the increased levels of many amino acids observed in this study.

In summary, a comparison of the metabolite profiles of RBCs infected with different strains of *P. falciparum* has revealed significant variations between strains, some of which associate with mutations in PfCRT. The information gained here sheds light on the biochemical changes brought about by mutations in PfCRT, and may provide insights into the elusive natural function of this protein.

## Online data

Supplementary data
