# Supplementary material for: 1H-NMR metabolite profiles of different strains of Plasmodium falciparum
Source: Biosci Rep. 2014 Nov 21;34(6):e00150. doi: 10.1042/BSR20140134 (PMC4240024; doi:10.1042/BSR20140134)

Figure S1. Aspartate signals from representative ^{1}H NMR spectra. The four high field aspartate signals from experiments performed with uninfected RBCs, co-cultured RBCs, and RBCs infected with each of the non-transfectant *P. falciparum* strains are shown. Aspartate was among the metabolites with the lowest intensity signals in the ^{1}H NMR spectra; the signal-to-noise ratios for this compound were typically > 6.

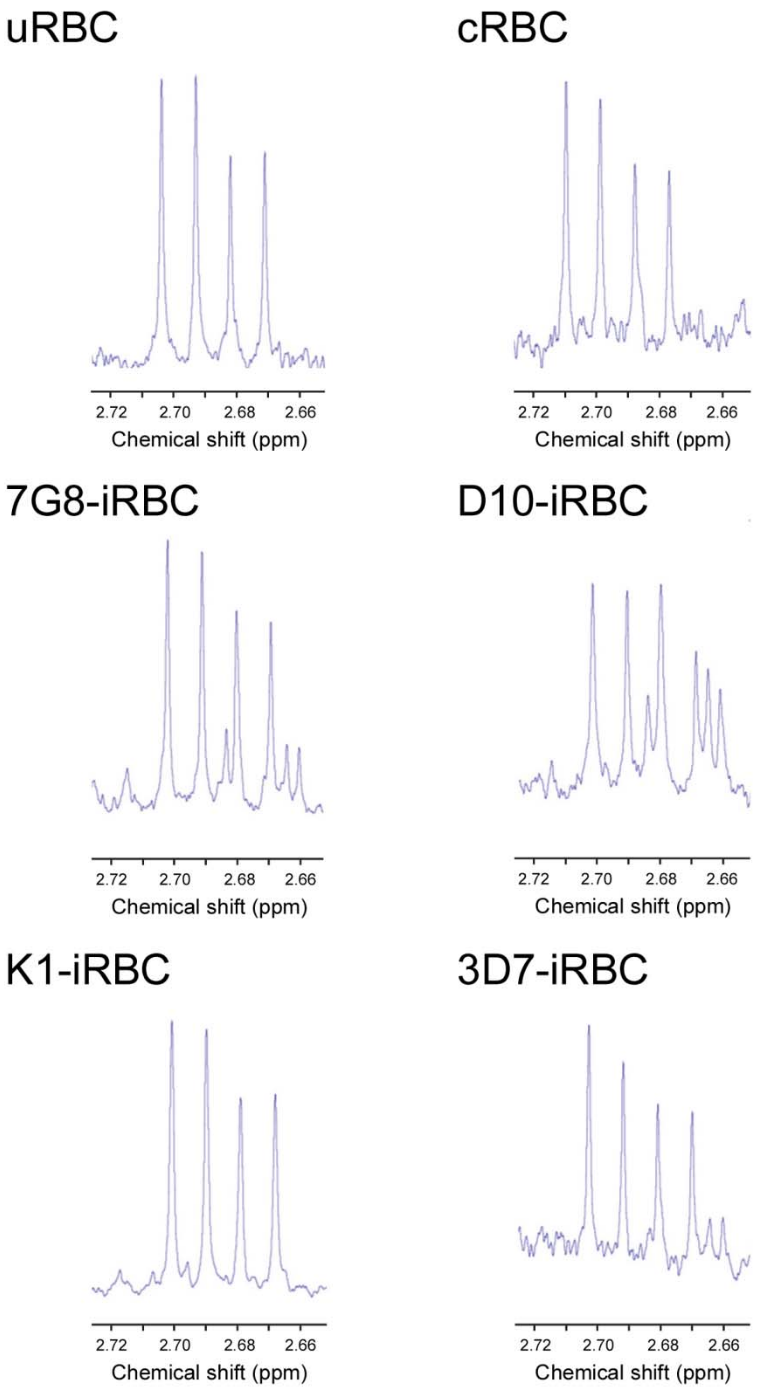

Supplement: Supplementary data [file bsr034e150ntsadd.pdf]
